# Cellular Behaviors of Periodontal Ligament Stem Cells in the Presence of Bone Grafting Biomaterials, In-Vitro Study

**DOI:** 10.3390/life13010089

**Published:** 2022-12-28

**Authors:** Vahid Esfahanian, Fatemeh Ejeian, Hajar Mohebinia, Zahra Sadat Zojaji Nejad, Maryam Yazdchi, Maziar Ebrahimi Dastgerdi, Mehrnoush Ebrahimi Dastgerdi, Mohammad Hossein Nasr-Esfahani

**Affiliations:** 1 Department of Periodontic, School of Dentistry, Isfahan (Khorasgan) Branch, Islamic Azad University, Isfahan 8155139998, Iran; 2 Department of Animal Biotechnology, Cell Science Research Center, Royan Institute for Biotechnology, ACECR, Isfahan 8159358686, Iran; 3 Department of Periodontics, Isfahan (Khorasgan) Branch, Islamic Azad University, Isfahan 8155139998, Iran; 4 Private Practitioner (Endodontist), Toronto, ON L3T0C9, Canada

**Keywords:** bone substitutes, cellular attachment, cellular proliferation, osteoblastic differentiation, periodontal ligament stem cells

## Abstract

Periodontal regeneration through the employment of bone substitutes has become a feasible strategy in animal and clinical studies. In this regard, we aimed to compare the periodontal ligament stem cell behavior in the vicinity of various bone grafting substitutes. Three types of popular bone substitutes, including allografts (Regen), xenografts (Cerabone), and alloplasts (Osteon) were studied in this experimental survey. The cellular attachment was assessed after four hours using the MTS assay and SEM imaging. In addition, cellular proliferation was investigated after 1, 3, 5, and 7 days through MTS assay. Osteogenesis was studied after 21 days of cell culture in a differentiation medium (DM+) and a normal medium (DM−), by employing real-time PCR and alizarin red staining. The highest cellular attachment was seen in the xenograft group with a significant difference in comparison to the other grafting materials. Despite the relatively low primary attachment of cells to allografts, the allograft group showed the highest total proliferation rate, while the lowest proliferation capacity was found in the alloplast group. Osteogenesis fount to be accelerated mostly by xenografts in both mediums (DM+ and DM−) after 3 weeks, while alloplasts showed the lowest osteogenesis. This study revealed that the type of bone substitutes used in regenerative treatments can affect cellular behavior and as a whole allografts and xenografts showed better results.

## 1. Introduction

In dentistry, guided regeneration of lost alveolar bone tissues has always been challenging, especially following a tooth extraction, periodontal diseases, or trauma. Periodontal regeneration describes employing bone substitutes and/or barrier membranes to guide cells for the growth and remodeling of lost tissues. Nowadays, the application of scaffolds for a wide range of cell-based therapeutic approaches (particularly based on mesenchymal stem cells) and protein/peptide-based (e.g., growth factors) treatments is offering intriguing debates in the field of periodontal regeneration [1].

In particular, according to Melcher’s theory, the type of presented cells around the tooth root during periodontal reconstruction determines the nature of forming tissue at the attachment region [2,3]. It is evident that periodontal ligament stem cells (PDLSCs) play a pivotal role in wound repair after any periodontal injury. They immediately are recruited to the lesion area, proliferate and differentiate into fibroblast cells that can accelerate the repair process. In addition, they are responsible for the secretion of various growth factors, which finally result in deposition of the extracellular matrix and have an immunomodulatory effect and profound impact on the differentiation of stem cells [4,5,6]. Indeed, recent studies revealed that the progenitor cells with the potential for regeneration of connective tissue are mostly located in the periodontal ligament [7,8]. These cells have favorable differentiation and greater osteogenic and adipogenic capacity compared to other mesenchymal stem cells (e.g., dental pulp stem cells and gingival stem cells) [9].

In tissue engineering strategies, proper attachment to the extracellular matrix, survival, and proliferation of desired cells are critical to achieve adequate growth and tissue repair [10,11]. The reconstitution of bone lesions is usually restricted to the employment of bone substitutes, including autografts, allografts, xenografts, and artificial alloplasts [12], with various degrees of success [13]. Some properties of scaffolds, including chemical and phase composition, porosity, pore size, and interconnectivity influence on passing cells and nutrients, which in turn affect proliferation, osteogenesis, and final tissue formation [6]. In this field, some studies have investigated particular commercial types of graft substances, and there are limited comparative studies between different types of grafts [14,15,16,17,18,19].

Therefore, this study was undertaken as a comparative study between three popular graft substances in periodontal regeneration, named allografts, alloplasts, and xenografts. We evaluated their cellular attachment, proliferation, and differentiation capacity, as the main parameters of tissue regeneration, using PDLSCs.

## 2. Materials and Methods

### 2.1. Materials Employed

All the materials were obtained from Gibco (Paisley, UK) unless otherwise stated. Demineralized Freeze-Dried Bone Allograft (DFDBA)/Freeze-Dried Bone Allograft (FDBA) allografts were purchased from Regen (ITB, Tehran, Iran), Osteon alloplasts from Genoss (Gyeonggi, Republic of Korea), and Cerabone xenografts from Botiss Dental (Berlin, Germany). MTS quantitative tests (Cell Titer 96^®^ Aqueous One Solution Cell Proliferation Assay, Promega, Leiden, The Netherlands) were carried out according to good manufacturing practice (GMP) procedures.

### 2.2. Culture and Proliferation of Periodontal Ligament Stem Cells

Previously characterized PDLSCs were cultured in a DMEM medium supplemented with 10% bovine serum, 1% Glutamax, and 1% antibiotic (300 mg/mL penicillin and 300 mg/mL streptomycin) at 37 °C in a 95% humidity and 5% CO_2_.

All the wells were coated with 200 µL of 0.1% agar to prevent cellular attachment to wells, except the control group of which PDLSCs were cultured on tissue culture plates (TCP). Therefore, 100 mg of allograft, alloplast, and xenograft particles were added and uniformly covered each well. To avoid future detachment, particles were initially washed twice with 500 µL of PBS (phosphate-buffered saline), and wells were kept at 4 °C for 24 h.

### 2.3. Assessment of Cellular Attachment and Proliferation via the MTS Assay

To assess cellular attachment, 5 × 10^4^ periodontal ligament stem cells were added to each testing well and incubated for 4 h at 37 °C in a 95% humidity and 5% CO_2_. Then, the wells were washed with the DMEM medium, and the MTS assay (Promega, Madison, WI, USA) was carried out to assess the number of cells attached to each well or on the particles, according to the manufacturer’s protocol. Briefly, a MTS solution in a culture medium (*v*/*v*: 1/10) was incubated with samples for 3.5 h under normal culture conditions. Then, the medium absorbance at 450 nm was measured by an ELISA plate reader (Fluostar Optima, BMG Lab Technologies, Ortenberg, Germany). The results were expressed as net absorbance versus uncultured samples.

For cellular proliferation assessment, MTS analysis was employed following one-, three-, five-, and seven-day incubations. The specimens were washed twice using PBS after each reading and incubated in the incubator until the next time point. Tests were performed two times with four separate tests (replications) for each stage.

### 2.4. Assessment of Cellular Morphology with SEM

A Field Emission Scanning Electron Microscope (FE-SEM; Hitachi S4160, Tokyo, Japan) was used to assess the morphology of cells adhered on the particles. Initially, samples were fixed for 2 h with 2.5% glutaraldehyde (Sigma-Aldrich, Saint Louis, MO, USA). Then, they were washed five times with double distilled water (DDI) for 20 min. Dehydration was carried out using 25%, 50%, 75%, and 95% ethanol for 5 min at each step. Finally, the samples were dried for 48 h in desiccators and, subsequently, placed on a carbon sheet. Grafting particles were coated with a thin layer of gold (15 nm thickness) by keeping them in a sputter coater (Hummer 2) for 20 min. 

Sample imaging was performed to study the surface morphology of particles and the cell morphology of the cultured PDLSCs after 24 h. To reach suitable confluency, 50,000 cells were plated on bone substitutes, and imaging was carried out after 24 h.

### 2.5. Osteoblastic Differentiation

PDLSCs were cultured on each sample at a density of 10^5^ per 12-well plate. After one day, the medium was replaced with a differentiation medium (DM+; DMEM medium supplemented with 10 mM beta glycerol phosphate, 10 nM dexamethasone, 50 mg/mL L-ascorbic acid, and 10% FBS) or normal DMEM (DM−). The medium exchange was performed two times a week up to 21 days of incubation. 

Finally, real-time PCR was performed for assessment of the expression of alkaline phosphatase (*ALP*) and secreted phosphoprotein 1 (*SPP1*, also called osteopontin or *OPN*) as the early and late markers of osteogenesis, respectively.

Additionally, alizarin red-S (ARS) staining was carried out for the assessment of calcification in the extracellular matrix. Briefly, each well was fixed with 96% ethanol solution for 15 min, washed with DDI and stained with 0.2% alizarin red (pH = 6.4) for one hour at room temperature. Subsequently, the unstained alizarin red solution was removed by washing with DDI, and absorbed ARS was solved in an acidic solution composed of 20% methanol and 10% acetic acid for 15 min. The net absorbance was measured using 450 nm vs. uncultured bone particles. 

### 2.6. Study of the Osteoblastic Differentiation Rate by qRT-PCR

In this test, the mRNA expression levels of genes relevant to bone differentiation were studied. For this process, first cells were collected by Trizol, total RNA was extracted, and cDNA was synthesized according to the manufacturer’s procedure. Finally, the expression levels of a housekeeping gene (*GAPDH*) and bone-related markers (*SPP1* and *ALP*) were assessed using qRT-PCR. Data analysis was performed through the ddCt method, in comparison to the gene expression level in untreated cells (day 0).

### 2.7. Statistical Studies

For all quantified experiments, data are presented as the means ± standard error of the mean (SEM). Statistical analysis was carried out by SPSS version 16.0, and one-way ANOVA was used to assess differences between experimental groups. The *p*-value of <0.05 was considered statistically significant.

## 3. Results

### 3.1. Surface Morphology and Attachment of Cells on Bone Grafts Substitutes

**Figure 1:**
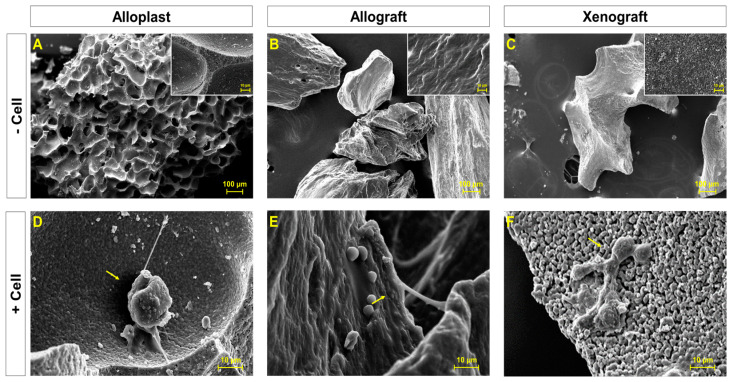
SEM imaging of bone graft substituents. Morphological features of uncultured alloplast (**A**), allograft (**B**), and xenograft (**C**) samples. Images with a higher magnification are shown as insets. Morphological characteristics of PDLSCs cultured onto alloplast (**D**), allograft (**E**), and xenograft (**F**) particles after one day. Cells are marked by arrows.

The surface examination of bone substitute particles by scanning electron microscopy (SEM) showed a highly microporous mode, with sharp angles in the alloplast structures (Figure 1A). Nevertheless, allografts and xenografts presented relatively smoother surfaces in micro-scale topography imaging (Figure 1B,C). As shown in insets with a higher magnification, alloplasts revealed quite a nanoporous surface. However, allografts were almost smooth with very small pores in some areas, while xenografts provided a medium roughness between the two modes.

Cellular attachment on the surface of particles was observed after 24 h in all three samples. In the alloplast and allograft samples, cells were not able to expand very well (Figure 1D,E), but a typical morphology of a PDLSCs on xenografts is shown in Figure 1F.

### 3.2. Assessment of the Attachment Capacity of PDLSCs on Bone Graft Substitutes

**Figure 2:**
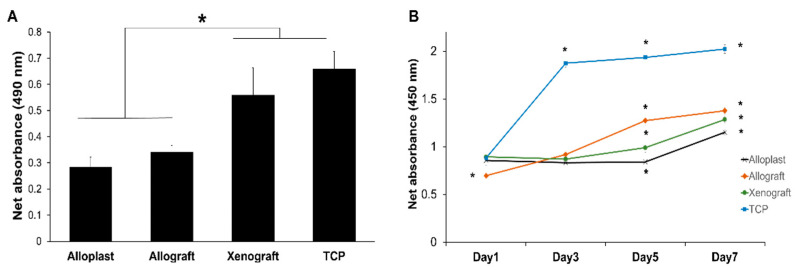
Adhesion and proliferation capacity of PDLSCs on bone graft substituents. (**A**) Primary attachment of cells on bone grafts, in comparison to the tissue culture plate after 8 h, assessed by MTS assay. * *p* < 0.05. (**B**) Proliferation analysis for PDLSCs cultured on bone grafts during one week, using the MTS assay. All data presented as mean ± SEM from three independent experiments.

Figure 2A shows the rate of cell attachment on the bone substitutes compared to that on the culture plate, 8 h post-seeding. Alloplasts (*p* = 0.009) and allografts (*p* = 0.002) revealed a significantly lower attachment capacity than the TCP group, while xenografts displayed a relatively similar potential for primary cell adhesion to the control group (*p* = 0.68). 

### 3.3. Assessment of PDLSCs Proliferation Rate on Bone Graft Substitutes

The overall growth patterns of PDLSCs on alloplasts and xenografts are similar, through which cells entered the exponential phase after five-day post-cultivation. Of note, the metabolic activity of cells in the xenograft group was significantly higher (*p* = 0.007). However, the cells grown on allograft left the exponential phase of growth on day 5 and entered the stationary phase (*p* < 0.001). Although the allograft revealed the least value on the first day, it reached the highest rate at the end of the following period. Cells cultured on the TCP displayed fast growth in the first three days and reached a higher steady-state value compared to the test groups.

### 3.4. Evaluation of PDLSCs Proliferation on Bone Graft Substitutes over Time

PDLSCs normally proliferated on the tissue culture plate and entered the plateau phase after three days. Meanwhile, cells with somehow similar absorbance on xenografts and alloplasts on the first day did not show notable cell growth before the third day in the case of xenografts and fifth day for alloplasts. This was followed by a slight (but significantly *p* < 0.001) increase until the seventh day. Despite the primary lower amounts of cells on allografts, they grew in the lag phase and reached the a significantly higher level of viable cells after seven days (*p* = 0.001) rather than the two other tested groups (Figure 2B).

### 3.5. Assessment of PDLSCs Bone Differentiation Potential on Bone Graft Substitutes

**Figure 3:**
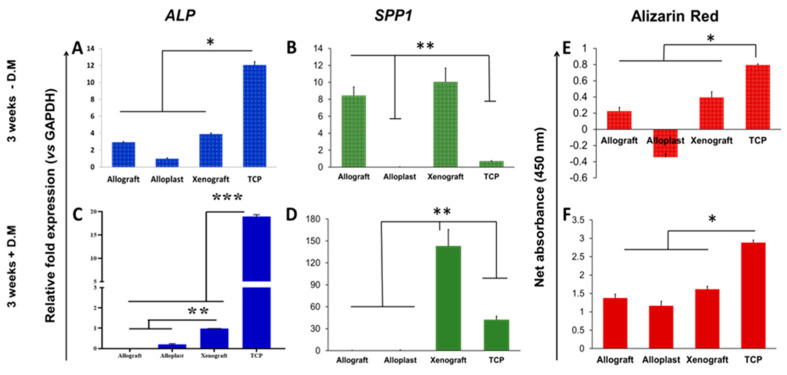
Evaluation of the differentiation potential for PDLSCs on bone graft substituents. The qRT-PCR analysis for measurement of the expression levels of *ALP* and *SPP1* after three weeks in the absence (**A**,**B**) and in the presence (**C**,**D**) of the specific differentiation medium. Quantification analysis of alizarin red staining for PDLSCs on bone grafts after three weeks in the absence (**E**) and in the presence (**F**) of osteogenic medium. * *p* < 0.05, ** *p* < 0.01, *** *p* < 0.001.

As shown in Figure 3A, in the absence of the differentiation medium, allografts and xenografts induced a moderate increase in *ALP* expression level (*p* < 0.05). On the other hand, PDLSCs displayed a considerable up-regulation of *SPP1* (*p* = 0.003) in contact with these materials, while both *ALP* and *SPP1* expression levels were significantly reduced in the alloplast group. As the control group, PDLSCs cultured on the TCP revealed a notable expression rate of A*LP* and a relatively low level of *SPP1* expression.

In the presence of a differentiation medium, *ALP* expression was negligible on allograft and alloplast substituents (Figure 3C). Although this value was significantly higher in the case of the xenograft group, it was markedly lower than control cells grown on the TCP (*p* < 0.001). As is obvious in Figure 3D, *SPP1* was expressed intensely in treated-PDLSCs cultured on xenografts, whereas no detectable expression of *SPP1* was found in the allograft and the alloplast groups in this circumstance. Even though the expression rate of *SPP1* in the TCP group was increased in comparison to that under the basic condition (without a differentiation medium), it was still lower than that in the xenograft group.

The results obtained from alizarin red staining after 21 days in the absence of the osteogenic medium (Figure 3E) showed a significantly higher raise in mineralization intensity in the TCP group than in xenograft and allograft groups (*p*< 0.05). However, in the presence of osteo-differentiation mediums, calcium deposition was elevated in all samples with the highest level in the xenograft group (Figure 3F).

## 4. Discussion

Recently, applying bone graft substitutes has received significant attention in the regeneration of osseous lesions and augmentations of hard tissue in periodontal treatments [20]. In addition to its routine application as a filler, an ideal bone graft has reasonable osteoinductive and osteoconductive potential [21]. Accordingly, in this study, three common allograft, xenograft, and alloplastic bone graft substitutes were selected due to their extensive application in the treatment of periodontal diseases. PDLSCs were introduced as the most important cell type in alveolar bone regeneration. Therefore, their regenerative responses to the different bone substitutes, which are used abundantly in clinical situations, have been evaluated.

The results of our study showed a significantly greater level of primary cell adhesion to xenografts rather than to allografts and alloplasts. It has been shown that the rougher surface topography and the higher porosity enhanced cell attachment to the surface of the particles [22]. In addition, the significant capillary property and suitable hydrophilic estate of xenografts are known as the determinative factors resulting in its higher attachment capacity [22,23].

On the other hand, a gradually enhanced cell proliferation rate was seen in the allograft group, which could be described by the better penetration of cells and growth factors through DFDBA. Our finding is consistent with the superior bone generation with DFDBA in clinical studies [24]. However, it has also been observed that the rate of proliferation and differentiation of stem cells in mineralized allografts was higher than that in the unmineralized ones. It is probably due to the omission of soluble osteogenic factors, such as BMP, from demineralized types [24]. In recent studies, it has been shown that when allografts have been used for filling bone cavities, the dimension of particles plays a big role in the attachment rate and proliferation of osteoblasts. In fact, higher densities of particles lead to decreased cellular attachment but increased cellular proliferation and differentiation [25].

The surface wettability and water uptake influence molecular movement and critical cellular behavior, such as proliferation [26]. Therefore, the discrepancy between the two materials could be described by different water absorption properties. In the case of xenografts, after primary wetting water absorption reaches the maximum level over a short time. The ceramic nature of these materials leads to brittleness and deformation at the contact interface over time [23]. On the other hand, different subtypes of allografts show gradual water absorption. In some kinds, the rate of absorbed water increases linearly with time [23]. In addition, the organic content leads to water absorption and swelling of the sample, which increases molecular movement in these substances.

The cell metabolic activity in the alloplast was almost constant during the first five-day post-seeding. This may be related to the synthetic origin and the biocompatibility of the material. In addition, the hydrophilicity of alloplasts is directly influenced by the porosities between granules and the crystallization rate of the grafting material [23]. Cellular proliferation was significantly lower in alloplasts due to lower attachment relative to the other two groups; however, allografts and xenografts showed no significant differences. 

Motamedian et al. showed low attachment of the dental pulp stem cells to the bone substitute scaffolds followed by a notable increase in cell−scaffold attachment during the processing time [27]. The results of the current study revealed a decrease in cell count after seven days, an increase in differentiation and size of cells, and better cell attachment and proliferation on TCP due to the rough surface of the scaffold.

### 4.1. Comparison of Osteoblastic Differentiation Rate between Allografts, Xenografts, and Alloplasts

The findings of the present study showed that on day 21 and in the absence of the differentiation medium *SPP1* and *ALP* gene expressions were significantly higher on xenografts and allografts than on the alloplast group, with no significant difference between xenografts and allografts.

In the presence of the differentiation medium, xenografts revealed higher *ALP* and *SPP1* expressions compared to the allograft and the alloplast groups, with no significant difference in the two latter ones. As a whole, these results can show the priority of xenograft and its precedence in the osteoblastic differentiation process. 

Platelet-derived growth factor (PDGF) changes bone regeneration through the increasing proliferation of osteoblasts, blocking differentiation of these cells and SPP1 and osteocalcin (OCN) incidence [28,29]. *ALP* is well-established as an initial marker of osteoblastic differentiation [30], which undergoes downregulation during the maturation phase of osteogenesis. The OCN and SPP1 are known as markers of the mineralization stage and their maximum expression occurs during days 14 to 28. They increase at the proliferation and final stages of osteoblastic development [24,27,30,31].

*ALP* and *SPP1* were expressed in all three bone graft groups on day 21, with significantly higher expression in the xenograft group. This discrepancy can be a sign of the precedence of xenografts in the differentiation process compared with the other two substances. Since the *ALP* is expressed at the beginning of the differentiation process, this difference can be a sign of passage from the initial differentiation stages in all three substances and it might be probably the higher peak of gene expression in xenografts. In addition, *SPP1* expression causes suppression of osteoblasts response and has a preventive role in osteoblastic activity [31]. Alizarin staining also confirmed higher mineralization in the xenograft group in comparison to the alloplast and the allograft groups.

### 4.2. Comparison of the Osteoblastic Differentiation Rate in the Presence or Absence of the Differentiation Medium

In all three groups, *ALP* expression was significantly higher in the absence of the differentiation medium (DM−) compared to in the presence of the differentiation medium. In the controlled culture, *ALP* activity was significantly inhibited on bone substitutes, showing that bone substitutes had both bioactive and biocompatible properties at the same time and they led to apoptosis of osteoblasts without contact with them [32]. Based on this finding, it seems the bone substituents can induce bone specification, but not final differentiation. Since the cellular proliferation rate in the osteogenic medium is less than in the standard medium, which is due to more rapid differentiation and earlier expression of osteogenic genes in the differentiation medium for allografts [27]. Deproteinization occurring in xenografts changed the spatial structure of substitute collagen, such as higher attachment, higher ALP expression, and matrix mineralization by the osteoblastic cells [33,34].

Growth factors are introduced as the critical regulators of differentiation mediums. One of these growth factors is PDGF, which causes chemotaxis and mitosis. It also has a synergic effect on cell proliferation when combined with allografts [35].

Our findings support the inherent potential of allografts for triggering bone specification, independent of any extrinsic factors due to its internal differentiation factors. However, xenografts and alloplasts better expressed *SPP1* located at the end of the differentiation spectrum in the presence of the differentiation medium. Based on the Alizarin red staining method, it was observed that the levels of osteoblastic differentiation on day 21 in all three experimental groups were significantly higher in the presence of the differentiation medium than in the absence of the differentiation medium. This may be attributed to the crucial role of inducing factors in the final osteoblastic differentiation and mineralization process.

In the absence of the differentiation medium, alloplasts had lower osteoblastic differentiation than allografts, but when the differentiation medium was added no difference was observed in the osteoblastic activity of the allograft and the alloplast groups. This might have happened because of the origin of these materials. While allografts have a biological origin, alloplasts are synthetic materials. Therefore, alloplasts need some extra help to induce differentiation.

In Miron et al.’s study, PDLSCs exhibited a suitable primary adhesion on calcium phosphate bone grafts; the expression of osteoblastic differentiation markers was much higher on the 7th day. Enamel matrix derivatives (EMDs) improved all outcomes and increased mineralization [36]. 

## 5. Conclusions

From this study: it can be concluded that the type of bone substitutes used in regenerative therapies can influence cellular attachment, proliferation, and differentiation. As a whole, allografts and xenografts showed better results in cellular attachment, proliferation, and differentiation, while the alloplast group was not comparable with these two groups. In addition, the osteoblastic differentiation process started earlier in contact with xenografts.

## Data Availability

Not applicable.
